# To Change or Not to Change: Perceptions and Experiential Knowledge of Tennis Coaches When Modifying Grip Technique

**DOI:** 10.3390/sports12120325

**Published:** 2024-11-27

**Authors:** Nicholas Busuttil, Kane J. Middleton, Marcus Dunn, Alexandra H. Roberts

**Affiliations:** 1 Sport, Performance, and Nutrition Research Group, School of Allied Health, Human Services and Sport, La Trobe University, 1 Kingsbury Drive, Bundoora, Melbourne, VIC 3083, Australia; k.middleton@latrobe.edu.au (K.J.M.); alexandra.roberts@latrobe.edu.au (A.H.R.); 2 Institute for Health and Sport, Victoria University, 70/104 Ballarat Road, Footscray, Melbourne, VIC 3011, Australia; 3 Performance Science, Research and Innovation, The Movement Institute, Melbourne, VIC 3042, Australia; 4 School of Energy, Geoscience, Infrastructure and Society, Heriot-Watt University, Edinburgh EH14 4AS, UK; marcus.dunn@hw.ac.uk

**Keywords:** coaching, modifying technique, constraints-led approach, physically-constraining tool, experiential knowledge

## Abstract

The purpose of this study was to explore the experiential knowledge of tennis coaches as it related to the development of grip positions in tennis athletes. Accredited tennis coaches (n = 11) completed semi-structured interviews consisting of open-ended questions about their coaching background, the importance of grip positions compared with other areas of foundational development, and their opinions on using physically-constraining tools (PCTs). Two major themes, “Grip positions are an adaptive skill” and “Why and how do I modify an athlete’s grip?”, were identified. Coaches expressed the opinion that grip positions were dynamic and a modifiable component of tennis stroke technique. Irrespective of shot type, grip positions were viewed as a non-negotiable aspect of talent development and intrinsically linked to other components of the stroke. Coaches questioned the necessity of technique refinement for grip positions given the complex and time-costly nature of bringing about effective motor-behaviour change. Some coaches expressed reservations about skill transfer into live match-play, intuitively expressing the concepts of the constraints-led approach to manipulate key variables within the athlete’s environment to foster learning. Future research should aim to assess the short- and long-term effects of PCT use in tennis and establish the extent to which PCTs can impact learning and skill transfer.

## 1. Introduction

Modifying technique can be difficult and typically requires a long period of time to achieve permanency [1]. It is an integral component of the role of a development coach, and, in many sports, being able to change an athlete’s “natural” technique is important for both performance enhancement and injury prevention [2,3,4]. Understanding coach knowledge and how it impacts coaching practices is a growing field of research. There is increasing interest in understanding why coaches make the decisions that they do within different contexts (e.g., [5,6,7,8]). It is well evidenced that coaches can provide substantial insight into the practical, performance-related questions that athletes and coaches themselves may need answering [9]. Coaches’ knowledge and their practices for skill development have been explored in different sporting domains such as swimming, gymnastics, and athletics [10,11,12]. While expert coaches predominantly use traditional skill development methods such as skill decomposition to reinforce “ideal” technique, they also intuitively manipulate constraints in their training environments to develop fundamental components of stroke technique [10].

Research has shown that coaches often express ideas consistent with the constraints-led approach (CLA) to skill acquisition [9,13,14]. The CLA provides a lens through which the complex interactions between environmental, performer, and task constraints can be explored [15]. Past research has reported that manipulating task constraints is a common practice among tennis coaches, demonstrating effective modifications for technique promotion in the serve [4] and scaling court sizes for improved match-play characteristics such as increased rally length and number of forehands played [16]. In addition, previous tennis research has primarily focused on aspects of movement including injury prevention biomechanics and physical preparation [17,18]. Grip position, or the way an athlete’s hand orients around their racket’s handle when executing a stroke, is a fundamental component of tennis performance [2,19,20]. Recently, tennis coaches, irrespective of experience level, indicated that “training grip positions” is a key part of foundational athlete development [19]. Grip positions are generally developed during the initial stages of skill development with guidance from the athlete’s coach [21], and have been shown to affect upper limb, racket, and ball kinematics in multiple strokes [22,23,24,25,26]. Specifically, the Eastern grip position has been associated with increased horizontal racket head velocity across the forehand compared with the Western grip [25,26], and in the single and double-handed backhand (non-dominant hand) compared with a non-preferred grip [22,23,24]. Specific grip positions have also been associated with both radial (Eastern) and ulnar (Semi-Western) wrist-sided injuries [20]. It has been reported that coaches consider it difficult to train grip positions, and that 65% of coaches would use a physically-constraining tool (PCT) to aid in this task [19]. The concept of “training” or “developing” specific grip positions alludes to the prescriptive coaching methods that have been dominant in tennis [3,4]; however, with a shift towards more athlete-driven methods [21], it is prudent to discuss ways in which physical (i.e., equipment-based) constraints can be used to drive technique change.

Despite the importance coaches place on grip development and the effects different grip positions can have on performance, there is little empirical evidence to guide coaches and practitioners as to how to effectively develop desired grip positions in developmental athletes. Interviewing coaches can provide insight into current practices and simultaneously explore potential avenues for further research. Specifically, the richness of interviews can provide a real-world basis to underpin future research that will be practically useful to coaches and other practitioners. This study sought to explore coaches’ perspectives on the importance of grip position, specifically during early tennis grip development. Further, we aimed to understand their current practices for technique change and explore their perceptions of the utility of PCTs.

## 2. Materials and Methods

### 2.1. Study Design

The following study was qualitatively descriptive in design and was approved by the La Trobe University Human Ethics Committee (#HEC20336), with written and verbal consent being obtained from each participant.

### 2.2. Participants

The cohort consisted of 11 accredited tennis coaches (male = 8 and female = 3; mean ± SD; 44.6 ± 13.7 years of age; and 19.7 ± 12.2 years of coaching experience). Participants were considered “accredited” coaches if they held an internationally recognised tennis coach certificate level 1 or greater (e.g., Tennis Australia Level 1, ITF level 1, or USPTA Level 1). All participants were above 18 years of age, were previously and/or currently involved in tennis coaching, and could understand and speak English. Participants were recruited initially through purposeful sampling and email distribution to national tennis organisations as well as social media. Snowball sampling was also used, encouraging coaches to forward the study’s information to their colleagues [27]. Participants are referred to by alphanumeric combinations (e.g., C1 represents Coach 1) and their demographics are shown in Table 1.

### 2.3. Interview Guide Development and Procedure

Semi-structured interviews were conducted using an interview guide (Table S1) consisting of open-ended questions. The interview guide was piloted with a tennis coach, a qualitative researcher, and a sports scientist. Following piloting, the interview guide was revised and refined by the research team to ensure clarity and cohesion. The data collection period was between 1 November 2020 and 30 January 2021. Participants were interviewed via an audio-recorded video conference (Zoom Video Communications, San Jose, CA, USA) by the first author (NB; mean duration: 54 ± 11 min). Before the interview, participants were provided with the information and consent form via email and were required to sign and return the consent form before an interview was organised. At the beginning of each interview, the participants were also asked to confirm consent before any questioning commenced. The interview was divided into three sections: (1) background information and demographics, (2) coach opinions regarding grip positions and stroke development with associated experiences, and (3) their opinions on the use of PCTs for grip-specific skill development in tennis.

### 2.4. Data Analysis

Open-ended questions were analysed using thematic analysis [28] using NVivo 12 (QRS International, Chadstone, VIC, Australia). Our analysis of participants’ experiences and behaviours was underpinned by a critical realist perspective. The authors recognise the “reality” that exists for coaches while acknowledging that our participants’ interpretation of reality is mediated by their own individual contexts. Our participants’ perspectives were taken at face value and interpreted as depicting the “truth” or reality of their experiences. Correspondingly, we used a largely descriptive method of thematic analysis to explore participants’ experiential knowledge [28] of developing grip positions in tennis players.

The familiarisation process began through verbatim transcriptions by the first author immediately upon completion of each interview using Microsoft Word, with three of the eleven interviews auto-transcribed (Otter.ai, Mountain View, CA, USA, V 3.44.0). Following each auto-transcription, the text was compared against the audio recording and corrections were made if required. Following familiarisation, data were coded by NB using a bottom-up process in which data were coded according to both descriptive elements of the data along with more theoretically informed ideas. Following review with AR, potential themes and sub-themes were identified from the codes based on clusters of meaning while keeping in mind the overarching research questions. Relevant data were then collated within sub-themes and, ultimately, themes. The dataset was then re-read to ensure that participant experiences and perceptions were adequately captured within the themes.

## 3. Results

Two themes were identified that contextualise the importance of grip positions in relation to foundational skill development. The themes were “Grip positions are an adaptive skill” and “Why and how do I modify an athlete’s grip?”.

**Themes**

### 3.1. Grip Positions Are an Adaptive Skill

Stroke development was considered to be a fundamental aspect of learning or developing tennis skills for nearly all participants, and the development of appropriate grip positions was perceived by all coaches as being an integral component of an effective tennis stroke. Coaches believed that an athlete’s technique and game style, including their grip position, should be developed on an individual basis. Coaches emphasised the importance of athlete self-discovery and the use of ”natural” grips when attempting to develop grip positions. They sought to engage their athletes in a collaborative process, with the coach’s role being to assist their athlete in determining the appropriate grip position and then develop their stroke around this decision.

**C9:**  “…I believe, more in natural grips, I believe more in terms of the player will find the grip that they like to play with… You know, so it’s about what does the player feel? What’s the natural grip? And how do we develop the biomechanics around that?”

The importance of athletes developing these natural grip positions through “*feel and comfort*” (C8) was emphasised, as it allows athletes to self-discover their preferred grip position in given strokes, scenarios, and to “*find the grip they want to play with*” (C9). Coaches noted that while there are specific ”grip positions” that athletes may adopt, there is variability and adaptation in the way that these positions are used.

**C8:**  “…grips are not always the same and even, you know, at least for the players that I talked to, they feel like they adjust just a little bit for slightly higher balls or a very low balls (sic). They need to make little adjustments when they play high volleys or low volleys, they make little adjustments for floaters…”

Coaches discussed that specific grip positions could be used in multiple shot types, and how that realisation impacted their coaching practices.

**C10:**  “…we wrote down all the things (strokes and playing scenarios) that were in a continental grip, and the things that weren’t. And it was like 17 or 18 that were (played with) in a Continental (grip)…it radically affected how I teach everything.”

While coaches believed strongly that grip positions are individual to the athlete and modifiable to the game circumstances, it was emphasised that a coach’s ability to assist an athlete who wished to change their “preferred” grip was important for effective match-play performance.

### 3.2. Why and How Do I Modify an Athlete’s Grip?

Modifying grip positions, as alluded to by the coaches interviewed, was considered a difficult process and must occur when the athlete’s chosen grip was outside of the coach’s range of acceptability and, thus, perceived to either increase injury risk or decrease performance. Coach 7 spoke of their observations from professional tennis, which is consistent with past research regarding professional players not using the Western grip for the non-dominant hand in double-handed backhands [29].

**C7:**  “(For the) forehand, (it) will be Eastern or Semi-western try to avoid anything, you know, too extreme (Western) either way. Because, you know, you look at the pro tour, like nobody’s really hitting forehands with a Continental or Western grip anymore. And try to avoid it. I mean, I have a very extreme Western grip, but I tried to just get them (students) not to go any further than Semi-Western.”

Coaches commonly expressed that there is a battle when developing a new grip position for specific skills; however, grip modification was deemed integral for skill longevity and progression.

**C7:**  “…with the age and the level of the athletes that I tend to coach, there’s a lot of, you know, when they change something…all of a sudden…they start to miss. And that’s when they just say, ‘oh, it doesn’t work, it doesn’t work’ and go back to what’s worked even though technically, it’s not great…Just getting them to see that…you will adjust, and you will get there. We’re trying to build you into a tennis player for the future.”

The lengthy process of grip change was dependent on an athlete’s intrinsic motivation to perform.

**C1:**  “It really depends on why they (are) looking at a grip change, if it’s something that is necessary for them to get to the next level or something that they already competent with but breaks down under pressure. It just depends on the why.”

An athlete’s choice of grip positions and associated stroke technique should be developed with a clear purpose, so that the athlete can relate and develop an understanding. This can be in the form of *“explaining the why*” (C2) behind each drill or linking the technical training to tactical situations and outcomes.

**C8:**  “I try to explain to them, so, whatever problems they encounter, I try to solve those problems. Like, when you change the grip, right, the ball starts to develop a lot of side spin on the serve, and also it goes more to the left.”

All coaches stated that they previously or currently use physical constraints to aid in changing an athlete’s grip, such as using specialty training tools or repurposing everyday equipment to encourage athletes to modify and maintain changes in grip positions. In particular, coaches used PCTs when developing the Continental grip, believing that they could aid in creating technique change. During the interview, Coach 10 retrieved and displayed a custom-designed modified tennis racket used to assist with the development of a Continental grip position for the serve.

**C10:**  “Oh, it’s (specialty tool) very effective…I want six of every size…(it is) absolutely fabulous to get them to understand (Continental grip) and then all you’re doing is showing them (while they use it), I think it’s fabulous…”

When developing adaptability in athletes’ grips, coaches were selective about terms used to promote technique change in their athlete’s grip position and overall stroke development.

**C9:**  “…we coaches need to be very, very careful about the words that (we) use. (The athlete) was being told to ‘brush’ the ball, ‘whip’ the ball, ‘get wristy’ with it. Just all sorts of things. And then naturally, her grip started to slip towards that (Western grip), because that’s the only way she could play the game and achieve what the coach was asking.”

It is worth noting in a discussion of “how” to change grip technique that coaches were adamant that while grip positions are important, their development was intrinsically linked to other aspects of tennis stroke development.

**C11:**  “I think the grip is intrinsically linked to the swing line and the stroke development of the player.”

Irrespective of shot type, grip positions were viewed as an essential aspect of talent development, and intrinsically linked to other components of the stroke. As a result, many coaches believed that grip position should be developed in conjunction with other stroke components and not in isolation. It is clear that coaches believe that changing the position in which an athlete holds their racket is an unavoidable process during foundational tennis development; emphasising the “why” behind changing the grip is an important consideration. When developing new grip positions or helping athletes to become adaptable in their use of their chosen grip, coaches use both physical tools and verbal instruction to change grips.

## 4. Discussion

The purpose of this study was to explore the practices of tennis coaches when developing grip positions during the fundamental development of tennis skills. These findings contribute to a greater understanding of the development of grip positions and coaches’ perceptions of best practices for teaching and modifying grip positions during skill development.

Grip positions were expressed by the coaches in this study to be dynamic and a modifiable component of tennis stroke technique. Coaches expressed the idea that playing tennis is chaotic and unpredictable, therefore, grip positions and stroke techniques should be developed to be adaptive in given scenarios and situations. One approach to understanding skill development, particularly within dynamic contexts such as sports, is the CLA [30,31,32]. Within the CLA, the interactions between individual, task, and environmental constraints facilitate the emergence of adaptive movement behaviours. In the context of nonlinear pedagogy, athletes will need to find their own performance solutions to satisfy the unique constraints imposed on them at the given time. Nonlinear pedagogy involves the manipulation that is aligned with the CLA to skill acquisition [31,32]. Commonly, coaches would associate constraints with the process of manipulating and/or modifying key variables in the performance and learning environment. From the current group of interviewed coaches, it was clearly articulated that the choice of appropriate grip is dictated by a variety of individual athlete factors and the specific shot type being played. The interaction between these factors creates the “*natural*” (Coach 8 and 9) grip for each athlete, which can then be adapted according to the specific task demands (i.e., shot and game context). Grip position can affect shot biomechanics in tennis, with impacts on shot performance (accuracy, ball landing coordinates, and ball speed) as well as upper limb and racket/club kinematics [22,23,26]. It is evident that different grip positions, both naturally developed and acutely modified, have different movement response options, and that individual athletes can adapt their movements and still achieve similar task success [22,23,24,26].

Coaches also described stroke development (i.e., learning different grip positions and associated stroke biomechanics) as being an important component of fundamental tennis skill development. Consistent with previous research, the coaches in this study described concepts aligned with constraints-based coaching [33]. A coach’s decisions and actions are highly contextual and are influenced by information from their knowledge/experiences, their environment, and the specific task at hand [5,15,34]. Language use was suggested to be an important consideration in effective grip and stroke development, a point that was exemplified by Coach 9 as they discussed the direct implications of coaching with instruction. Coach 9 stated that instead of changing athlete behaviour through instructions, coaches should design training to facilitate functional changes in athlete behaviour (in this case, designing training to prompt change in grip positions). As expressed by coaches, grip positions and stroke development should be considered linked and complementary components of a player’s development as each grip position has specific movement characteristics across a range of strokes [3,4,22,23]. Grip positions are interrelated with stroke development, so the athlete should not develop such components away from critical sources of information within the performance environment. For example, the speed, spin, and trajectory of the incoming ball provide informational points from the environment for the receiving athlete to respond to effectively (returning the ball with a desired task outcome), and the response can be constrained (or enhanced) by their grip position in use and movement responses. In the context of tennis, it is important to develop an adaptive player who can use grip positions in varying match scenarios and contexts through exploiting the interacting components in the performance environment.

“Effective” athlete development can have alternative meanings for different coaches, which can be dependent on their professional development, practical experience, and own interventions. However, coaches agree that the process of grip modification is difficult and should be attempted only when there is a specific performance or injury rationale behind the change. Coach 1 detailed the importance of both coaches and athletes understanding the “why” of grip change for an athlete, and questioning whether a technique refinement process for grip positions is necessary. In high-level athletics, technique refinement is a consistent practice largely orchestrated from a coach’s own experiences with constraint manipulation; it acknowledges that the process for creating desired movement outcomes or behaviours is done subtly and over an extended period of time [35]. The CLA to coaching encourages athletes to find their own solutions to the problems posed by the constraints of their sport at a given time [14]. As demonstrated in these interviews, tennis coaches intuitively understand the concept of constraints and manipulate key variables within the athlete’s environment to foster learning. This purposeful modification of task and environmental constraints allows coaches to create scenarios in which learners can explore various options to solve performance problems and create their own individual solutions. The comment from Coach 1 in the current dataset suggests that athletes and/or coaches still adopt drills and interventions to refine techniques, even though the fundamental skill has been learnt. Such interventions in relation to grip positions involve using forms of physical constraints. The process of grip modification for coaches usually occurs when an athlete is using an “*extreme*” grip (Coach 7), more commonly known as the Western grip. The Western grip is avoided by coaches, particularly in the forehand and double-handed backhand, as it is not a desirable stroke technique, which aligns with previous research findings. Specifically, the Western grip is perceived by coaches as undesirable due to players’ limitations on faster surfaces. It was found that development-level coaches rarely teach the Western grip during foundational stages [19], while it was reported that in the top 100 professional players in both the men’s and women’s competitions, no athletes used the Western grip [29]. It is appropriate to suggest that the continual adaptation and modification of technique over time is an unavoidable and non-linear process, and one which can be conceptualised through the CLA and the manipulation of task constraints [36].

One such example of a constraint spoken about by these coaches is the idea of physically-constraining tools—devices or objects that encourage athletes through physical constraints to maintain a certain grip position, simultaneously allowing athletes to explore new movement solutions with task success while maintaining the desired biomechanical technique. Coaches alluded to these ideas, describing the athlete’s self-discovered grip as being the most optimal, while also acknowledging that some grip positions can be injurious and so should be avoided. Physically constraining athlete movements (using tools or other methods) for specific task outcomes is a common practice [37,38], however, it is generally only used in short interventions. Facilitating short-term interventions could be applied with the use of a PCT specific for grip positions. As Coach 8 expressed, the importance of being able to adapt grip positions in live match-play is something that athletes like and subconsciously do. When asked about using a PCT for grip-specific skill development, it seems the most appropriate course of action would be to utilise it in either the earliest foundation years of stroke development [19] or as a transition tool if facilitating the later stages of skill development, as expressed by the coaches in the current study. Inherently using PCTs creates the understanding of the “feel” of movement coming from either the ball impacts during the racket/ball interaction and an awareness of components within their stroke technique (e.g., timing and swing velocity) during the early stages of movement preparation. Athletes may be afforded the opportunity to explore movement solutions with the tool through the inherent mechanisms of tactile information and by generating intrinsic and kinaesthetic feedback. Through these mechanisms, this may allow the athletes to develop self-organised perceptual-motor abilities to achieve effective movement outcomes [37]. In the example of tennis, athletes self-regulate their movement to effectively return the ball to the opponent. For coaches, intervening with a grip-specific PCT for early foundational development for grip positions and stroke technique, or facilitating a grip modification process during late-stage skill development, may be most appropriately done with either the gradual removal, or with an acute exposure to using a PCT, under different practice and feedback settings [39]. Implementing this approach may ensure that athletes receive adequate exposure to a PCT in part and encourage them to autonomously coordinate their movements according to the newly desired techniques outlined by the coach, thereby preventing athletes from developing a reliance on the tool. This method may enable athletes to promptly execute these actions within the dynamic context of live match-play, creating a training environment that closely simulates actual performance conditions and, possibly, fostering enhanced skill development. The current findings detail that coaches do view grip positions and stroke technique as interacting components for skill development, rather than each being in silos. Grip positions were also expressed as being a dynamic, adaptable skill to enable effective performance during live match-play. This emphasises the importance of developing tennis skills along with the ability to be flexible and modifiable, both of which are characteristics closely aligned with the requirements of tennis match-play.

### Limitations and Future Directions

These results may be limited by the varying backgrounds of the coaches who participated in the semi-structured interviews, specifically given the range of years of coaching tennis (2 to 39 years). It can be assumed that a 37-year difference in coaching experience and the subsequent variety of environments experienced would have an impact on coach perceptions [33]. Specifically, the variety in lived coaching experience would indicate that their own development as a coach and athlete, and, subsequent, their teaching/coaching styles, would likely be different [40]. This may have implications for the interpretation of the questions during the interviews, with the coaches’ responses likely being a product of habitus, that is, the way that an individual has learned to perceive and act in the world based on previous experiences, both as an individual athlete and practicing coach [41]. Despite the range in coaching years, all coaches had experience coaching athletes from grassroots, or the beginning of an athlete’s tennis development at the time of data collection, suggesting that they were actively involved in foundational tennis development, which was the target phase for this study’s research question.

Importantly, coaches indicated that they would use a PCT to assist with training grip positions if one were available. While a number of these tools exist commercially, to the authors’ knowledge, there are no reliable studies indicating their effectiveness in tennis or any other racket, stick, or club sport. These findings, specifically those regarding the use of PCTs, need to be further explored within an applied environment: one which attempts to answer queries posed by coaches. Future research should focus on determining the performance effects of using PCTs, and to better understanding their impact on skill and technique development.

## 5. Conclusions

This study investigated the perceptions and experiential knowledge of tennis coaches when teaching grip positions. The current research identified the complex nature of grip positions, grip technique modification, and PCTs with two major themes, “grip positions are an adaptive skill” and “why and how do I modify an athlete’s grip?”, that embrace the major contexts associated with developing grip positions in tennis. Coaches expressed that grip positions are intrinsically linked to stroke and technique development, and that grip positions during competition are adaptive skills. The necessity of changing grip technique was questioned by coaches, given that the process is lengthy, enduring, and athlete-dependent. Irrespective of the coaches’ background and certification level, all coaches expressed that they would use a PCT for grip-specific skill development, however, this enthusiasm was caveat by reservations about the transfer of the new ”grip skill” to live match-play. From these findings, it appears that coaches may consider the choice of grip positions as an adaptive skill rather than being a prescriptive technical component of tennis performance, and they would be open to methods for making changes occur faster and more effectively. Future research should aim to determine the short- and long-term effects of PCTs for sport-specific tasks, and their transfer into and subsequent impact on performance. Further, these findings open the avenue for future research to explore how and when tennis players adapt their grip in response to different training and competitive settings.

## Figures and Tables

**Table 1:** Demographic information about the 11 coaches, detailing their age (years), highest attained coaching certification, number of years coaching with a certification, and the level of coaching experience.

	Age (Gender)	Highest Coaching Certification(s)	Years of Coaching	Coaching Experience
Coach 1	40 (M)	USPTA Elite Professional (Level 3), Tennis Australia High-Performance (Level 3), ATP and WTA certification	25	Grassroots through to elite
Coach 2	38 (M)	USPTA Elite Professional (Level 3), Tennis Australia High-Performance (Level 3), ATP and WTA certification	24	Grassroots through to elite
Coach 3	24 (M)	Tennis Australia Club Professional (Level 2)	8	Grassroots through to semi-professional
Coach 4	58 (M)	Tennis Canada Instructor Course (Level 1)	8	Grassroots and social
Coach 5	41 (F)	Tennis Australia Junior Development (Level 1)	5	Grassroots and social
Coach 6	63 (M)	ATPCA Performance Tennis Pro (Level 3)	30	Grassroots through to semi-professional
Coach 7	30 (F)	Lawn Tennis Association Tennis Instructor (Level 2)	2	Grassroots and social
Coach 8	59 (M)	Slovenian High-Performance Certification (Level 3), USPTA Elite Pro	35	Grassroots through to professional
Coach 9	28 (M)	Tennis Australia Club Professional (Level 2)	14	Grassroots through to semi-professional
Coach 10	63 (F)	USPTA Elite Professional (Level 3)	39	Grassroots through to semi-professional
Coach 11	47 (M)	Tennis Australia High Performance (Level 3)	27	Grassroots through to professional

## Data Availability

The original contributions presented in the study are included in the article, and further inquiries can be directed to the corresponding author.

## Supplementary Materials

The following supporting information can be downloaded at: https://www.mdpi.com/article/10.3390/sports12120325/s1, Table S1: Interview guide used during the data collection process.
