# Supplementary material for: To Change or Not to Change: Perceptions and Experiential Knowledge of Tennis Coaches When Modifying Grip Technique
Source: Sports (Basel). 2024 Nov 27;12(12):325. doi: 10.3390/sports12120325 (PMC11679087; doi:10.3390/sports12120325)
Supplement: Supplementary file 1 [file sports-12-00325-s001.zip › sports-3287377-supplementary.pdf]

Table S1. Interview guide used during the data collection process.

QUESTION	PROBES	STIMULI	PURPOSE
**Background information**			
- Please tell me about your coaching background – how did you get into tennis coaching?<br>- When did you decide you wanted to be a coach?	- Formal coaching education?<br>- Length of time coaching?<br>- Levels/ages?<br>- School/university?<br>- Playing level?	- Level of comp/results<br>- Rankings of students/self<br>- Courses completed.	- To place all future responses in context and establish current coaching level and experience.
**Section 1: Current stroke development coaching methods.**			
- What do you associate with the term stroke development?<br>- What does the term grip training mean to you?<br>- What methods do you currently use to develop technique and grip postions?<br>-	- This is what stroke development means to me…<br>- This is what grip training means to me…Considering this…(linking in grip positions)<br>- What aspects do you focus on most when trying to develop a stroke?	- Concepts<br>- Processes<br>- Age groups<br>- Materials used<br>- Forehand/backhand/serve<br>- Based on individual ability?<br>- Effective communication? Reinforcement of "effective/correct technique?	- To familiarise the coach with terminology for stroke development and grip training.<br>- To understand what the coaches think are important practices for stroke development and how important grip positions/training is to them.<br>- Definition of effective stroke development and grip training by coach.

- What grip positions are you suggesting to your students to use?<br>- And for what strokes?	- Forehand, 2-hand backhand serve<br>- Does this change based on age/gender athlete experience?<br>- Do you take a student's progress/improvement into account when training grip positions?	- Drills, racket and ball speed<br>- Performance under pressure (match-play scenarios)<br>- Just tell them? "here use this"?<br>- Do you explain how the grip works?<br>- What counts as improvement/progress?	- To know the current practices of grip positions in tennis strokes.<br>- To understand what measures are used to assess what an "effective grip transition" is.
- What are the biggest hurdles/ barriers you face when trying to develop tennis strokes?	- Student effort?<br>- Discomfort?<br>- Not demonstrating "fast improvement"?	- Child not purposely moving their hand around the grip's handle?	- To understand what may inhibit stroke development and training of grip positions.

- Also for grip positions?<br>- How important do you think training grip positions in tennis is for stroke development?<br>- **Rank 1-10, 10 being most important.**	- Engagement<br>- Students remembering the grip positions?		- Need to use answers.<br>- To determine the magnitude of importance of training grip positions in tennis strokes.
**Section 2: Understanding the coach practices and opinions for grip positions, grip development.**			
- When developing a student from an early age, how would you rank the importance of grip training for overall student development appose to the following aspects.<br><br>- Rank 1-5, 5 being most important.	- Physical conditioning<br>- Technique development<br>- Court movement<br>- Transferring concepts from training to match-play<br>- Grip positions<br>- Use survey answers for probes	- Strength, speed, agility, flexibility training<br>- Biomechanics, arm movements<br>- Recovery steps, running to the ball<br>- Why do you think these other components are so important?	- To see where they rank the importance of grip training amongst other tennis related requirements, and to see if this is still from the survey.
- What do you look for to say that "this student has	- Would this change with age /experience/stroke?	- Psychological?<br>- Competition scores?	- To know specific, preferably measurable

effectively changed grip positions”?<br><br>- How long do you typically spend training grip positions of tennis strokes?<br>- Is there anything that you have implemented to help with grip training for students?<br>- What are your methods of measuring effective grip change?<br><br>- What are the key components that you employ during a developmental lesson.	- What do you think makes an effective grip change?<br>- Does this change with strokes?<br>- What strokes do you find this most difficult in?<br><br>- Previous athletes you have done this on?<br>- **Draw on experience.**<br>- Specific examples of the methods.<br><br>- How do you structure it?<br><br>- What order?<br><br>- Does this change depend on the above mentioned	- Training efforts?<br>- Student compliance<br>- Visuals of ball trajectory?<br>- Spin/speed?<br><br>- Mini tennis?<br>- Basket feeding?<br>- Live hitting?	factors that tennis coaches use to assess effective grip change.<br><br>- To determine what coaches do to help train grip positions?

- In other interviews, coaches have mentioned that …. Is important for grip training. What are your thoughts on this?		Use previous examples/literature examples to guide questions<br>- Developmental age<br>- Ability to see progress	
- Do you think that using a grip learning tool (ie grip mould) would be effective for improving grip training?<br>- Do you think coaches would use this tool?<br>- Do you think students would use this tool?	- Yes/no, why/why not?<br>- *show tool*<br>- Would it help them to not move their hands?	*Explain what the mould will achieve, how it will be constructed and what it's purpose will be.*<br>- Individualised moulded finger guiding, should promote finger grip strength, and<br>- Same diameter as a standard base grip	- To know a direct opinion of using a grip mould device
Is there anything else you would like to add?			
